# The “Smart Dining Table”: Automatic Behavioral Tracking of a Meal with a Multi-Touch-Computer

**DOI:** 10.3389/fpsyg.2016.00142

**Published:** 2016-02-11

**Authors:** Sean Manton, Greta Magerowski, Laura Patriarca, Miguel Alonso-Alonso

**Affiliations:** Laboratory of Bariatric and Nutritional Neuroscience, Center for the Study of Nutrition Medicine, Beth Israel Deaconess Medical Center, Harvard Medical SchoolBoston, MA, USA

**Keywords:** eating behavior, food choice, meal, automatic tracking, multi-touch computing, human-computer interaction

## Abstract

Studying how humans eat in the context of a meal is important to understanding basic mechanisms of food intake regulation and can help develop new interventions for the promotion of healthy eating and prevention of obesity and eating disorders. While there are a number of methodologies available for behavioral evaluation of a meal, there is a need for new tools that can simplify data collection through automatic and online analysis. Also, there are currently no methods that leverage technology to add a dimension of interactivity to the meal table. In this study, we examined the feasibility of a new technology for automatic detection and classification of bites during a laboratory meal. We used a SUR40 multi-touch tabletop computer, powered by an infrared camera behind the screen. Tags were attached to three plates, allowing their positions to be tracked, and the saturation (a measure of the infrared intensity) in the surrounding region was measured. A Kinect camera was used to record the meals for manual verification and provide gesture detection for when the bites were taken. Bite detections triggered classification of the source plate by the SUR40 based on saturation flux in the preceding time window. Five healthy subjects (aged 20–40 years, one female) were tested, providing a total sample of 320 bites. Sensitivity, defined as the number of correctly detected bites out of the number of actual bites, was 67.5%. Classification accuracy, defined as the number of correctly classified bites out of those detected, was 82.4%. Due to the poor sensitivity, a second experiment was designed using a single plate and a Myo armband containing a nine-axis accelerometer as an alternative method for bite detection. The same subjects were tested (sample: 195 bites). Using a simple threshold on the pitch reading of the magnetometer, the Myo data achieved 86.1% sensitivity vs. 60.5% with the Kinect. Further, the precision of positive predictive value was 72.1% for the Myo vs. 42.8% for the Kinect. We conclude that the SUR40 + Myo combination is feasible for automatic detection and classification of bites with adequate accuracy for a range of applications.

## Introduction

Selecting food from a range of options is a key characteristic of the way humans eat, particularly in environments where food is abundant and easily available (Menzel and D'Aluisio, 2007). Deciding what to eat represents a recurring dilemma and a common experience throughout an individual's life (e.g., family meals, college dining halls, workplace cafeterias, supermarkets, restaurants etc.). It has been estimated that an average person in the United States makes 200–250 food-related decisions per day (Wansink and Sobal, 2007) and purchases food in supermarkets that carry, on average, ~45,000 items (Food Marketing Institute, 2014). Food choice and eating behavior ultimately determine dietary patterns and nutritional intake and thus influence the risk of chronic diseases such as obesity, diabetes, cardiovascular disease, and certain cancers. A better understanding of eating behavior and food choice can bring new insights to stimulate the development of novel therapies and public health interventions to facilitate healthy eating.

Studying how people eat is not short of limitations and challenges. In fact, eating is one of the most complex human behaviors, and most methods of analysis that exist nowadays are indirect and rely on self-report (e.g., questionnaires). Quantitative approaches in the laboratory have largely focused on the meal, the natural unit of eating in humans (Meiselman, 2000). A better understanding of the behavioral dynamics of a meal can provide ecologically relevant information: the interplay between homeostasis, sensory aspects, reward and cognition. At a basic level, the act of eating triggers a sequence of physiological and behavioral events that ultimately lead to suppression of further eating until the next episode (the so-called “satiety cascade”; Blundell, 1991). Research here typically involves experimental evaluation of food intake before and after single-course meals (Blundell et al., 2009).

An important milestone in this field was the development of the Universal Eating Monitor (UEM) by Harry Kissileff (Kissileff et al., 1980). Via continuous weighing of a single plate with the use of a hidden scale, the UEM provides direct, accurate information of what occurs during the meal (also called meal microstructure or meal microanalysis), e.g., parameters such as eating speed or bite size. Since its description, 35 years ago, the UEM has been used in a number of applications related to obesity and eating disorders, including the evaluation of new medications (Kissileff et al., 1986; Laessle et al., 2007; Dovey et al., 2009; Halford et al., 2010; Schulz and Laessle, 2012).

However, this and closely related methods (Ioakimidis et al., 2009) do not allow the study of *ad libitum* eating when multiple options of food are available, i.e., multiple plates. More recently, there have been a few approaches to examine this dimension, based on combinations of food weighing and video recording (Allirot et al., 2012; Nornberg et al., 2014). Other alternatives include virtual reality simulations (McBride et al., 2013), or the use of food replicas (Bucher et al., 2012). Despite remarkable progress in technology—in particular, recent advances in wearable sensors, interactive platforms and ubiquitous computing—applications to the study of eating behavior remain largely underexplored, with some exceptions in the realm of ingestive and bite sensors (Sazonov et al., 2009; Fontana and Sazonov, 2013; Scisco et al., 2014). Detection of bites with a wrist-worn gyroscope can achieve 95% accuracy for a single-plate meal and 86% in free-living conditions (Dong et al., 2012; Scisco et al., 2014) and can also be used to induce slower eating rates (Scisco et al., 2011).

The existing methods to quantify eating behavior and food choice in humans are useful and valid and have made significant contributions to the field. However, there is still room for improvement, particularly in laboratory studies that require not only capturing but also manipulating complex aspects of the meal, such as cognitive influences, situations, and contexts. Importantly, there are no methods yet that allow full automatization, online analysis and interactive capabilities. Integrating contemporary technological advances into the field can open new research directions and contribute to a better and more precise understanding of how humans eat. The aim of this study was to examine the feasibility of a new computerized system that can be used in the context of a meal environment to evaluate eating behavior and food choice in an automatic and interactive manner.

## Overview of the multi-touch system and application to the case of a meal

The Samsung SUR40 is a tabletop computer running the Microsoft Surface platform. It includes multi-touch capabilities that are powered by an infrared camera behind the screen. The Surface software development kit (SDK) provides access to the raw image stream (a stream being a continuous inflow of data, in this case image frames from the infrared camera) recorded by this camera. We explored the possibility of leveraging this raw image stream to perform automatic detection of intra-meal eating behavior and food choice in a multi-plate format. The advantages of such a methodology lie in the possibility for on-screen feedback and interaction to be presented in response to food choice and bite rate during a meal.

The process of automatic recognition of intra-meal food choice was broken into two distinct phases: bite detection and bite classification. Bite detection is the phase of recognizing when a bite has been taken, regardless of the source plate. We explored a number of methods to perform bite detection, using different sensors and algorithms. Bite detection then triggers the process of bite classification, using the SUR40 raw image stream to determine the source plate of the bite. A three-plate experiment was designed to test bite detection and classification together. After analysis of the results of the three-plate experiment, we decided to attempt to refine the bite detection methods in a single-plate experiment, using a Myo armband with a nine-axis accelerometer as an alternative approach.

## Experiment 1: three-plate meal (bite classification and bite detection testing)

### Bite classification

The Surface SDK provides functionality for recognizing 256 unique “byte tags,” black squares of a specified size with white circles in specific patterns. These tags can be printed and attached to the bottom of objects so that their position can be tracked while on the SUR40. This byte tag functionality was used to track the position of the center of each plate.

**Figure 1:**
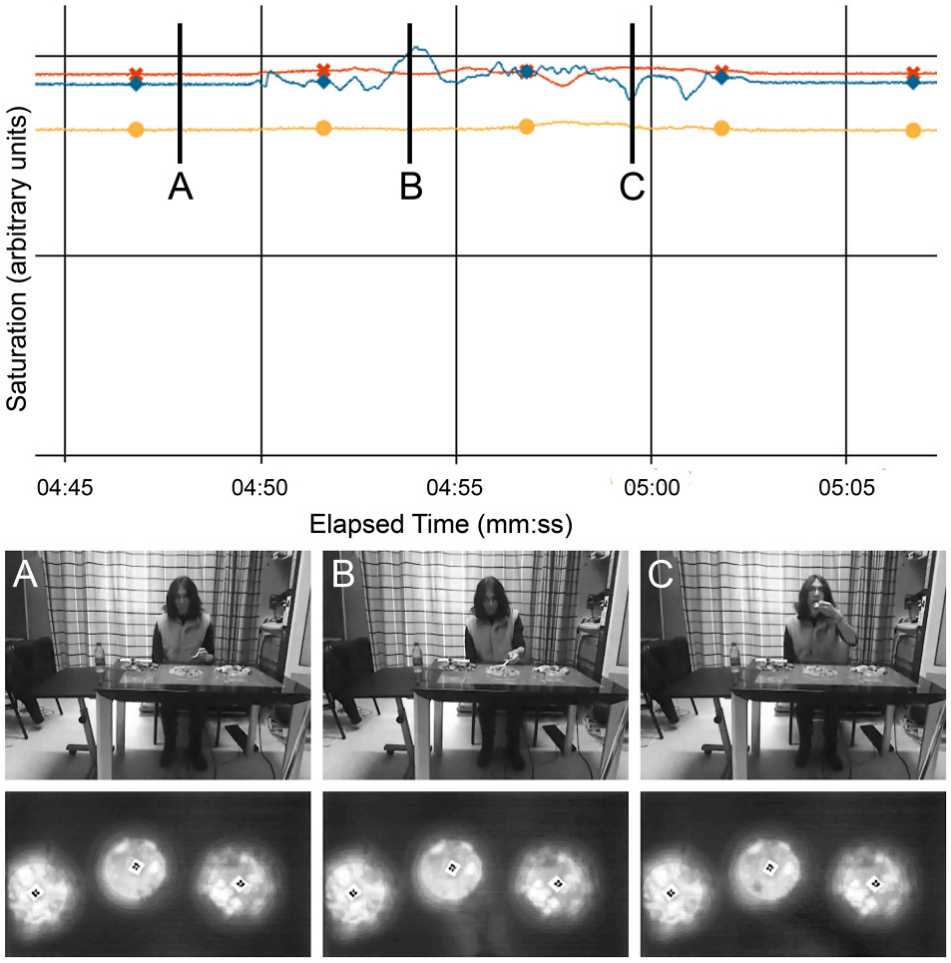
**Comparison of saturation measurements with Kinect and SUR40 videos**. The blue line with diamond markers, which shows the most fluctuation, corresponds to the central plate in the video frames. The frames are labeled (A–C) corresponding to the times of the vertical markers in the graph. Frame A takes place before the start of a bite—note that the saturation remains steady while there is no interaction with the surface. Frame B takes place as the food is being picked up with the fork. Note that the saturation has reached a peak here, corresponding to the increase in brightness in the bottom-right area around the plate, as visible in the SUR40 frame. Frame C takes place as the food is being placed in the mouth. Note that there is a slight shadow as compared to Frame A in the region to the bottom-right of the plate, corresponding to the local minimum.

In each frame, the grayscale saturation of the raw image was calculated for a square with its top-left corner (or top-right for left-handed eaters) at the detected position of the tag and extending 2 inches beyond the border of the plate. The grayscale saturation is a value ranging from 0 for pure black to 255 for pure white, for each pixel and corresponds to the infrared intensity captured by the camera. The calculated value was the average saturation within the square region. It was observed that when a bite is taken from a plate, there are two phases of distinct changes in the saturation value. First, as the arm comes close to the screen to take food from the plate, the saturation rises above baseline as the arm causes a greater infrared reading. Then, as the arm is raised up to take a bite, the saturation falls below baseline, as the arm, when a little further from the screen, casts a shadow compared to the baseline infrared levels of the lighting in the room (bright white fluorescent bulbs directly overhead). These stages are illustrated in Figure 1.

Thus, the prototype was designed around the hypothesis that whenever a bite was detected, it could be attributed to the plate which had the greatest overall flux (average of absolute value) within a short window preceding the bite. The window used was 30 frames, or ~0.5 s, as the SUR40 raw image stream has a framerate of 60 fps (some jitter in the framerate means that 30 frames does not always equal exactly 0.5 s). Clear plastic plates with a diameter of 10 inches were used, conferring the advantage of additional flux changes being detectable when the hand is directly over the plate or food is removed from the plate.

### Bite detection

The SUR40 was paired with a Kinect to provide video recording of the experiments for manual verification of bites and also with the hope that the Kinect could be used to detect the gesture of a bite, while the SUR40 classifies the source plate. Microsoft has developed proprietary algorithms for combining the color and depth streams of the Kinect (image streams with color frames, or grayscale frames representing the calculated distance of each pixel from the Kinect, respectively) to provide “skeleton-tracking,” the detection of various key points along the body. Skeleton tracking is optimized for detecting poses in a standing position, but the Kinect SDK also allows for a seated tracking mode where only the points along the upper half of the body are tracked. The seated mode was used for obvious reasons when utilizing the skeleton-tracking feature during the experiments. Initially, it was hoped that bite detection could be performed by using a lower threshold on the distance between the position of the hand and head points. However, it was found that the detected hand point would undergo significant jitter between frames when moved too close to the face, usually jumping away from the face point and the actual hand position. Thus, it quickly became apparent that the use of the skeleton-tracking feature alone would not suffice to perform bite detection.

An alternative method was developed, combining the color image stream and the skeleton stream. The shoulder-center and head points of the skeleton stream were observed to be less jittery than the hand point. These positions were used to extrapolate an area of the color stream which should correspond to the region surrounding the subject's mouth. The subject was equipped with a utensil of a high contrast color, i.e., a lime green plastic fork, and in each frame, the program calculated the RGB distance of each pixel to the target color of the utensil. In computing, colors are defined by an RGB (red, green, blue) value, where any color can be represented as a combination of different intensities of red, green, and blue. The RGB distance is defined as the distance between RGB values if they are mapped in Cartesian coordinates, i.e., red, green, and blue are three orthogonal axes. Thus the RGB distance is calculated as:

$$\sqrt{{\left(R_{2}-R_{1}\right)}^{2}+{\left(G_{2}-G_{1}\right)}^{2}+{\left(B_{2}-B_{1}\right)}^{2}}$$

A lower threshold was set so that only pixels within a certain RGB distance of the target color would be considered color-matched.

The threshold was set such that only the fork was capable of triggering color-matched pixels to be detected (for the lime green color we used, an RGB distance threshold of 30 was able to do so reliably). The number of color-matched pixels in the frame was then tallied, and if this sum exceeded a certain threshold, then a bite detection was triggered. After the threshold was initially exceeded, further bite detections could not be triggered until a certain number of frames after the number of color-matched pixels fell back below the threshold.

The Kinect runs at a speed of 30 fps, and a minimum separation between bites of 10 frames was used, or 1/3 s. While it would be unreasonable to assume that a subject would take many bites in such quick succession, the rationale for choosing such a low threshold was, initially, to prioritize sensitivity (i.e., not missing any bites) over reduction of false positives.

During pilot testing, it was quickly apparent that this color-matching method was more reliable than the pure skeleton-tracking-based approach. However, upon observation of some initial recordings, it was discovered that many bites still went undetected, because with certain rotations of the hand while bringing the fork to the mouth, there was not enough surface area of the fork exposed to the Kinect for the green pixels to be detected. The less surface area facing the Kinect, the greater the chance that what was visible of the fork in the frame would be obscured by reflections from the lighting in the room. Thus, the use of the green fork was abandoned in favor of a green wristband. The wristband provided the advantage of having more consistent visibility regardless of the rotation of the hand. If it resulted in reliable bite detection, it would also confer the advantage of being able to run experiments with finger foods or sandwiches.

### Manual verification

Bite detections were scored as true positive (TP), false negative (FN), or false positive (FP). Bites were considered TP when the system recorded a bite detection (or FN if it failed to record one) within the duration of the arm being raised or lowered in taking a bite. Any detections falling outside these visually verified time windows, or duplicate detections within a single window, were marked as FP.

Given that bites are events which occur at arbitrary points along a continuous timeline, they cannot be considered as discrete points which can be classified in a binary manner as bite/not-bite; as such, there is no way to quantify true negatives. The accuracy of bite detection can thus be gauged using the measures of sensitivity/recall, i.e., TP/(TP+FN), and precision/positive-predictive-value, i.e., TP/(TP+FP), but not specificity. Classification accuracy is then determined as the percentage of correctly classified bites out of the total number of TPs.

### Subjects and experimental procedures

**Figure 2:**
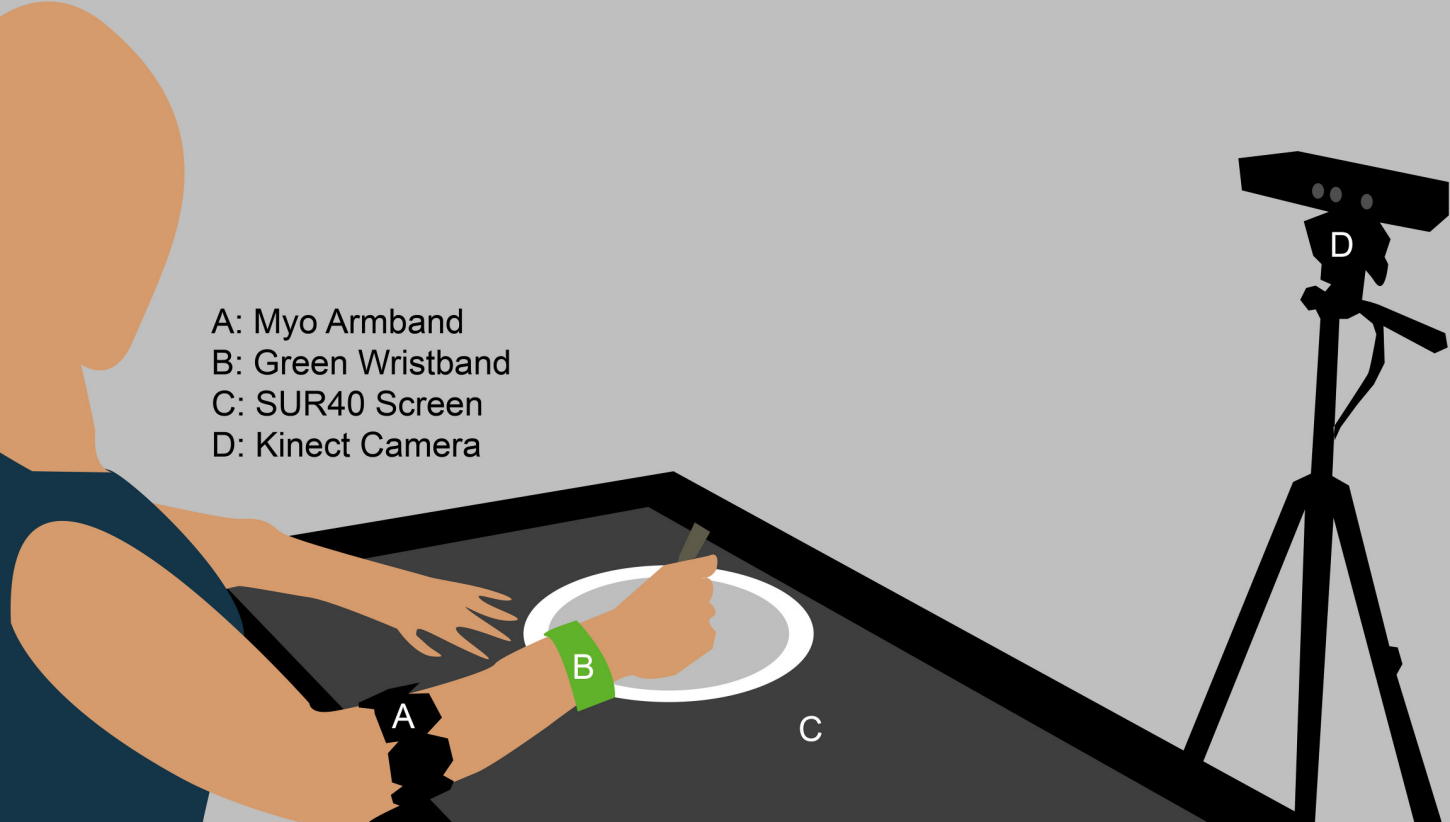
**Mockup of the experimental setup, shown with only one plate**. The distance of the Kinect to the subject varied across our tests. To perform skeleton tracking, it must be placed further from the subject. Otherwise, when used for manual verification purposes or to monitor the position of the green wristband, it can be placed closer to the subject to capture more detail.

Five healthy, lean subjects were tested during a lunch-time meal under at least 6 h of fasting. The subjects were aged 20–40, one female, and were volunteers recruited from local campuses of the Boston area. They signed an informed consent which explained that the experiment was to test a system for automatic food choice tracking during a meal. Study procedures were approved by the Institutional Review Board of Beth Israel Deaconess Medical Center. The three plates used in the meal were assigned to salad, fruit, or entrée (chicken and rice, or tofu and black beans for the 1 vegetarian subject). Each was filled with an excess of its respective dish to ensure that it did not run out before the subject reached satiation. The program settings were adjusted to associate the correct byte tag with each plate. Subjects were also given a water bottle, placed on the left side of the table. Drinking events were not automatically tracked. All subjects were right-handed, so the green wristband was placed on the right wrist. See Figure 2 for a mock-up of the experimental setup. They were instructed to focus only on eating and were required to turn off their mobile phones at the beginning of the session. Participants were asked to only take bites with their right hand and only with the provided utensils, and use their left hand to use napkin, drink water and perform other non-eating movements. Additionally, they were asked not to move the plates as much as possible, and to refrain from mixing foods from multiple plates in one bite.

The program was then launched and recording started (Kinect color video stream, Surface raw image stream, calculated saturation for each plate and the green pixels detected per frame were all logged). Plates were placed on the screen one by one. A display on the screen was used to confirm that each plate was properly detected and that the regions where saturation is tallied were well spaced apart. The display also showed the Kinect feed and was used to confirm that skeleton-tracking had been initiated successfully.

This display was then hidden, and the subject was instructed to eat as naturalistically as possible until satiated. The testing room was quiet and distraction-free, and the subject was left alone for the duration of the meal. When finished, the subject pressed a button to close the program and end the recording.

### Results

Manual verification using the Kinect videos counted 320 total bites across the subjects. The number of bites falling into each category is shown in Tables 1, 2. The resulting measures of overall accuracy were 67.5% sensitivity/recall and 64.1% precision/PPV for bite detection. Bite classification accuracy came out to 82.4%. These are the values calculated using the total number of bites in each category across subjects, rather than the mean value of the results within each subject's experiment (also shown in Tables).

**Table 1:** **Three-plate experiment bite detection results**.

**Subject**	**TP**	**FN**	**FP**	**Total Detections = TP + FP**	**Total Actual Bites = TP + FN**	**Sensitivity (Recall) = TP/(TP+FN)**	**Precision (PPV) = TP/(TP+FP)**
1	62	11	27	89	73	0.849	0.697
2	21	60	5	26	81	0.259	0.808
3	31	8	52	83	39	0.795	0.373
4	56	24	32	88	80	0.700	0.636
5	46	1	5	51	47	0.979	0.90w
Total	216	104	121	337	320	0.675	0.641
Mean	43.2	20.8	24.2	67.4	64	0.716	0.683
St. Dev.	15.3	21.0	17.8	25.0	17.57	0.246	0.180

**Table 2:** **Three-plate experiment bite classification results**.

**Subject**	**Correctly Classified**	**Misclassified**	**Total = TP from Detection**	**Accuracy = Correctly Classified/Total**
1	50	12	62	0.806
2	18	3	21	0.857
3	31	0	31	1.00
4	49	7	56	0.875
5	30	16	46	0.652
Total	178	38	216	0.824
Mean	35.6	7.60	43.2	0.838
St. Dev.	12.2	5.82	15.3	0.113

The sensitivity and precision varied greatly from subject to subject, with some (e.g., subject 5) having their bites detected consistently and with low FPs, and others showing very poor performance on sensitivity (e.g., subject 2) or precision (e.g., subject 3). This inconsistency is reflected in the high ratio of the standard deviation for each measure. It may have resulted from differences in the gesture used by each subject to take a bite, variations in the contrast of the wristband to the subject's clothing, or differences in the skeleton tracking performance for each individual. Overall, the bite detection performance (sensitivity in particular) was less than desired.

**Figure 3:**
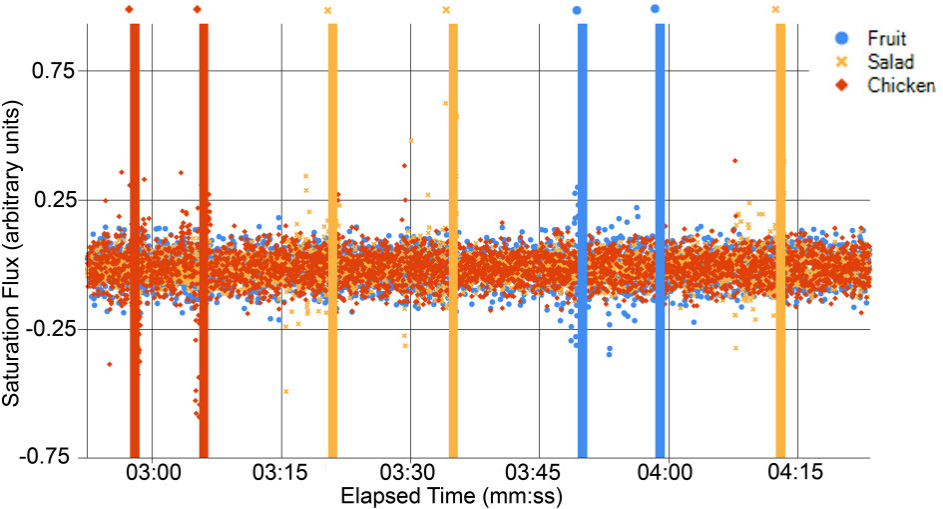
**Graph of the flux for each plate**. The manually-verified time of actual bites taken are marked by the vertical lines of the same color, with a marker included below each line as well, to clarify when viewing in grayscale.

However, the bite classification performance was more satisfactory, as well as consistent across subjects. Figure 3 illustrates how the saturation flux of each plate works as an excellent classification mechanism; the fluxes stay within a baseline range for the most part, but before each bite, the flux associated with the correct plate has points very obviously above and below the normal baseline.

## Experiment 2: single-plate meal (bite detection testing)

### An alternative method for bite detection

After the less than optimal sensitivity performance in the three plate experiment, we decided to perform another experiment using only a single plate in order to focus exclusively on bite detection performance. Additional logging was added of the detected shoulder-center and head positions, in order gain better insight into the performance issues, as only the analyzed green pixel detection was logged in the prior experiment.

Not convinced that this insight would necessarily help us adjust the settings to gain better performance from the Kinect, we also implemented an alternative detection method. The Thalmic Labs Myo armband is a consumer device designed to perform gesture detection of a limited number of gestures, primarily through electromyography, but also using a nine-axis accelerometer. We used the Myo for its convenient access to this nine-axis data, as opposed to the gesture recognition provided by its SDK.

**Figure 4:**
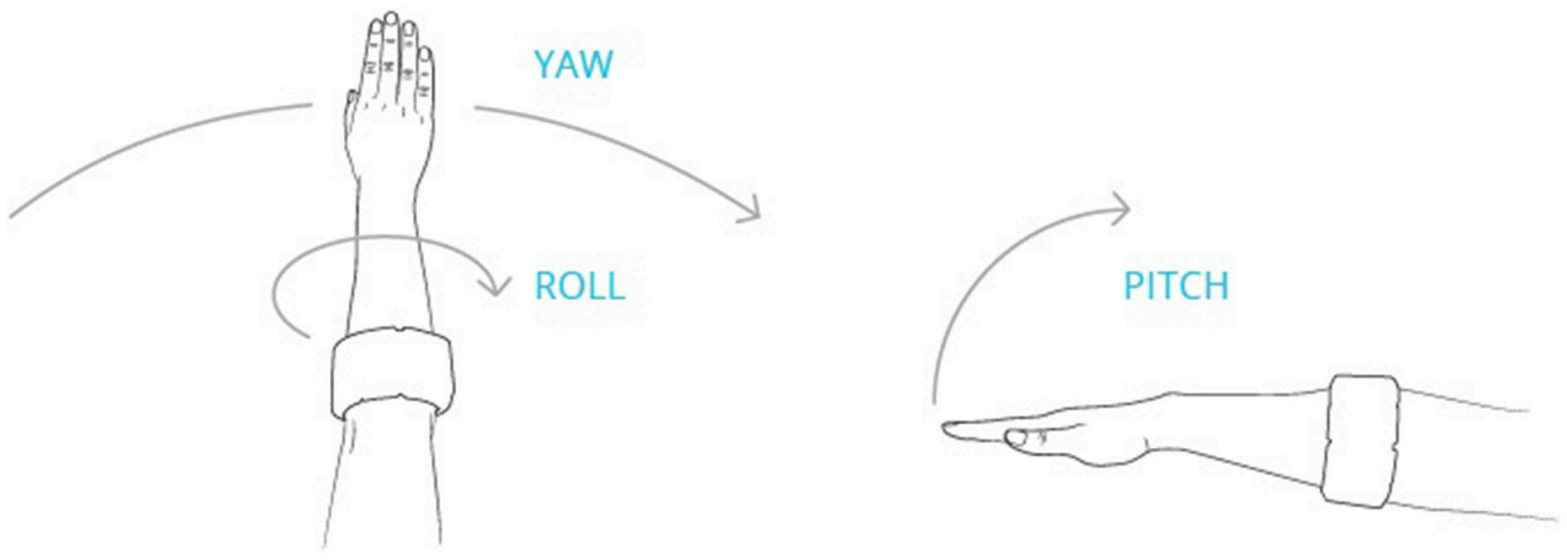
**Illustration of how pitch, yaw and roll are measured in relation to the orientation of the Myo Armband**. Pitch, yaw, and roll are orthogonally independent; thus pitch will reflect the elevation of the arm, regardless of the orientation of the hand, i.e., the hand does not need to remain palm down as shown in the sketch. (Source: http://developerblog.myo.com/gui-without-g-going-beyond-screen-myotm-armband/).

A nine-axis accelerometer consists of an accelerometer that measures acceleration in three axes, a gyroscope that measures the rotation around those three axes, and a magnetometer, which measures orientation in 3 axes relative to magnetic north but is calibrated to output data relative to the main axes of the Myo. These are referred to as pitch, roll, and yaw (see Figure 4). The pitch in particular is useful, as it essentially represents a measure of the incline of the arm relative to a flat surface.

During a bite gesture, the pitch will always increase as the arm is raised to the mouth and decrease as the arm is lowered to the plate. Thus, we hypothesized that a simple upper threshold on the pitch could be used to trigger bite detection. The application was programmed to log all nine axes of data so we could search for other potential correlations that might be useful for bite detection. Bite detection with the Myo was also restricted to the same number of minimum frames between bite detections as the Kinect, with retriggering also suppressed until after the pitch measurement fell back below threshold.

### Subjects and experimental procedures

In the single-plate experiment, the same five subjects were tested at lunch-time under similar conditions. Subjects wore the green wristband from the previous experiment on their right wrist, and the Myo on their right forearm. Byte tags were not necessary and saturation data was not logged for this experiment. In addition to the Myo nine-axis data, the Kinect skeleton tracking data for the head and shoulder-center joint positions was logged in these sessions, as opposed to only the calculated green pixel count. The program display was used to verify that Myo data was being received and that the Kinect had initiated skeleton tracking. The display was then hidden as before. A single plate was placed in front of the subject on the surface, containing the same food and amount as the subject's entrée plate in the three-plate experiment. The subject was given the same instructions as the previous session.

### Results

Manual verification of the videos found a total of 195 bites across the subjects. As presented in Table 3, The Kinect had an overall sensitivity of 60.5% and precision of 42.8%, compared to 86.2 and 72.1% for the Myo. The Myo results were obtained using a pitch threshold of 0.25.

**Table 3:** **Single plate experiment bite detection results**.

**Subject**	**Kinect**					**Myo**				
	**TP**	**FN**	**FP**	**Recall**	**Precision**	**TP**	**FN**	**FP**	**Recall**	**Precision**
1	19	18	25	0.514	0.432	35	2	4	0.946	0.897
2	16	16	25	0.500	0.390	14	18	3	0.438	0.824
3	10	24	25	0.294	0.286	30	4	23	0.882	0.566
4	33	19	71	0.635	0.317	49	3	34	0.942	0.590
5	40	0	12	1.00	0.769	40	0	1	1.00	0.976
Total	118	77	158	0.605	0.428	168	27	65	0.862	0.721
Mean	23.6	15.4	31.6	0.588	0.439	33.6	5.4	13	0.842	0.771
St. Dev.	11.1	8.14	20.3	0.233	0.173	11.6	6.44	13.2	0.205	0.164

**Figure 5:**
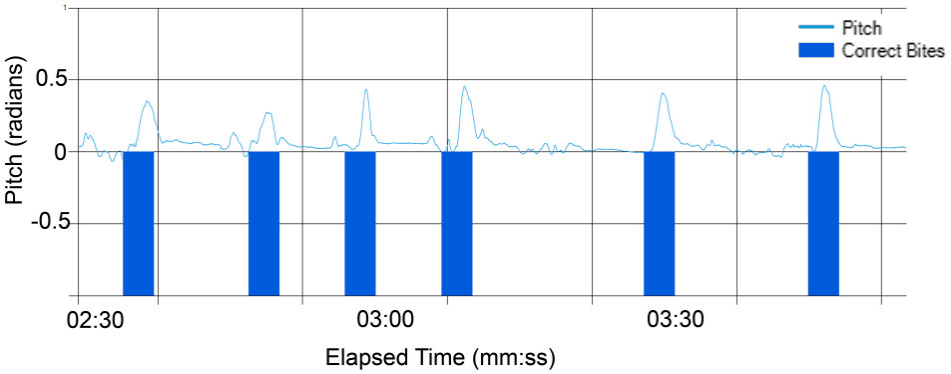
**Graph of pitch readings along with vertical markers of manually verified bite times**.

The Myo showed significantly better performance than the Kinect. Figure 5 illustrates how cleanly the pitch peaks line up to the manually recorded time of bites. Other axes of the nine-axis data were examined, and while some possessed certain visually noticeable correlations, they were all much noisier than the pitch data. Thus, the pitch was verified to be not only the most intuitive measure, but the easiest to analyze.

Examination of the Kinect logs revealed that there were significant amounts of jitter in the detected shoulder-center and positions. This jitter was not as bad as that of the hand point, as described in our initial exploration of skeleton-tracking based bite detection. However, the jitter of the shoulder-center and points was still major enough to make them unsuitable reference points.

While the Myo bite detection was generally good across subjects, it performed very poorly for Subject 2, with only 43.8% sensitivity. Examination of the videos revealed that while other subjects tended to remain mostly stationary in their torso position, raising the fork up all the way to their mouth, Subject 2 generally moved their head downwards most of the way to meet the fork, thus raising their arm far less and leading to a lower pitch peak for each bite.

## Discussion

The bite classification accuracy came out to 82.4% in the three-plate experiment and in the single-plate experiment, the Myo showed 86.2% sensitivity and 72.1% precision. The combined results of the two experiments suggest that bite classification by the SUR40 combined with bite detection by the Myo can be used as a first-of-its-kind system for automatic, online analysis of intra-meal food choice with interactivity possibilities. It is appreciated that a completely ideal system would have even greater sensitivity, precision, and classification accuracy, but we believe the obtained levels should be adequate for a wide range of applications, especially given that there are no other published methods offering the same capabilities. The closest alternatives are the experimental restaurant system (Allirot et al., 2012) or the Intelligent Buffet (Nornberg et al., 2014), but these methodologies still rely on manual coding of bites and do not offer a fully programmable and interactive meal table environment. Better sensitivity, in the order of 94%, can be obtained for bite counting via tracking of wrist motion but limited to a single-plate meal, and 86% for the case of uncontrolled free-living conditions (Dong et al., 2012). Our performance data is in the range of this second scenario.

The feature of automatic, online analysis running on a full Windows operating system opens up an almost unlimited range of possibilities for intra-meal experiments studying the effects of feedback or interactions based on the subject's eating behavior. From a programming perspective, it would be fairly trivial to integrate feedback such as bite counters and visualizations of bite rate to be displayed directly on the tabletop, allowing the subject to remain more naturally engaged in the meal than if they had to consult a separate screen or device. More complicated feedback such as intake estimates for calories or macronutrients would require more programming but still be quite straightforward to integrate into the system.

Many types of interactions could also be introduced. Experimental manipulations such as stimuli or tests could be introduced to the meal, with high temporal resolution. These could take place at set intervals (enabling the study of contextual influences) or be triggered by the online analysis (for instance, after a certain number of bites or above a certain bite rate). These manipulations would make it possible to study a wide range of questions for which there are currently no available methodologies. There is also great promise in terms of development of therapeutic interventions. Potential interventions could range from more passive, e.g., simple visual or auditory feedback, to extremely engaging, e.g., gamification (the process of introducing game mechanics to motivate certain behaviors, i.e., objectives and rewards associated with optimal eating behaviors). Whether passive or engaging, interventions could also occupy a spectrum from very minimal (i.e., non-intrusive feedback, simple reward system) to completely changing the eating experience (i.e., immersive video or music feedback, gamification with a strong narrative aspect). An additional application could be the design of computerized neuropsychological tasks requiring ingestion or selection of certain foods as part of performance. Altogether, the combination of online tracking, a fully programmable environment and interactivity can open a whole new way of designing experiments at the intersection of human-computer interactions and human-food interactions.

We believe that the breadth of new potential applications, combined with the relatively naturalistic eating experience, is a worthwhile trade-off for the less-than-perfect level of accuracy. In consumer research or applications, the accuracy may well be sufficient. In scientific studies requiring higher degrees of accuracy, manual verification can be used instead to obtain the final results. However, beyond the capability of interactions during the experiment, the automation could still be quite valuable for screening purposes or when quick decisions are needed during the course of an experiment. When running an experiment with a very large subject pool, the automatic analysis could be used as a preliminary way to observe trends in the data before undertaking the time- and labor-intensive task of manual verification on that scale.

Furthermore, it is important to note that these findings represent the first stage of development of this methodology. The algorithms for bite detection and classification that were used are simplistic, and use of more advanced techniques such as machine learning and computer vision algorithms can only improve the accuracy in future iterations.

As more subjects are studied, it will also be possible to refine the method to account for different styles of taking bites. As was discussed in the single-plate experiment, the sensitivity was extremely low for Subject 2, as they lowered their head and torso to meet the fork much more than the other subjects. To improve future performance in such cases, subjects could be instructed to focus on raising the fork all the way up, without bringing their head down to meet it, though this would interfere with the aim of having the subjects eat as naturalistically as possible. Another alternative is to exclude subjects who lean down too much when taking their bites. However, the best possible solution could be to develop a calibration procedure where the subject would be asked to take a few bites to first verify the ideal pitch threshold for their individual bite gesture style, before proceeding with automated detection.

Even though the method we describe has major advantages for automatic detection of eating behavior in terms of food choice and bite rate, a limitation is the lack of estimation of bite weight. This parameter can be detected with the use of so-called mechanistic approaches, which monitor weight and provide a measure of gram per minute (Blundell et al., 2009). In our case, the estimation is an event over time, but the bite size can only be estimated through a division of total amount of food taken divided by the amount of bites. This might not be entirely optimal, as it has been shown that throughout the course of a meal there is a gradual decrease in the bite size that reflects changes in subjective appetite. Even though we did not explore the following idea, it will be possible, in the future, to design methodologies that integrate new technologies for continuous monitoring of food volume and weight contained in plates.

Another limitation that we should acknowledge is that our method is designed for laboratory studies and thus it does not allow for tracking of eating behavior in free-living conditions. For such applications the use of portable technologies such as bite counting or ingestion sensing devices seem more suitable (Sazonov et al., 2009; Fontana and Sazonov, 2013; Scisco et al., 2014). Other disadvantages of our methodology are limited tabletop computer size, high cost (at least $7000 for the tabletop computer), and factors which may interfere with natural eating behavior. Both the use of the armband and other technology, as well as the fact that the objective of the study was explained in the informed consent could have caused subjects to modify their eating behavior. Eating under observation is known to reduce food intake in laboratory studies when subjects are aware that they are being observed and is a general limitation in the field (Robinson et al., 2015). In our case, the technologies used may have heightened this awareness. A strategy to account for this effect in the future would be within-subject experimental designs with multiple conditions in randomized order.

Here subjects were instructed to eat only during the sessions, using solid foods that were easy to grasp with a fork, and clear plastic plates with fixed dimensions to fit comfortably within the tabletop dimensions. While we were successful in implementing the technology capacity to automatically track meal behavior in this context, future studies should also evaluate how this system could perform in other scenarios, e.g., with a variety of foods, a range of containers and utensils or when eating occurs together with other behaviors, including social interactions.

Lastly, the methodology we describe here was developed on a SUR40 platform, a multi-touch computer that was discontinued from the market in 2013. However, other brands of Microsoft Surface tabletop computers now exist, including ones much larger than the SUR40, which could enable our methodology to be expanded to more plates or even multiple subjects in a social eating context.

## Conclusion

In this study, we developed a new methodology, providing, for the first time, automatic and online detection and analysis of eating behavior and food choice during a meal. This method uses the combination of orientation data provided by a Myo armband and analysis of the raw image stream from a SUR40 computer. While the system does not currently achieve perfect accuracy, its promise lies in the wide range of potential applications, given the system's capability of online analysis and interactivity.

## Author contributions

Concept design: SM, GM, MA. Software development: SM. Data Acquisition: GM, LP. Data analysis: SM. Primary manuscript writing: SM, MA. Additional manuscript review: GM, LP.

### Conflict of interest statement

The authors declare that the research was conducted in the absence of any commercial or financial relationships that could be construed as a potential conflict of interest.
